# What Is the Incidence of Intracranial Bleeding in Patients with Mild Traumatic Brain Injury? A Retrospective Study in 3088 Canadian CT Head Rule Patients

**DOI:** 10.1155/2013/453978

**Published:** 2013-07-15

**Authors:** C. E. Albers, M. von Allmen, D. S. Evangelopoulos, A. K. Zisakis, H. Zimmermann, A. K. Exadaktylos

**Affiliations:** ^1^Department of Emergency Medicine, University Hospital Bern (Inselspital), University of Bern, 3010 Bern, Switzerland; ^2^Department of Neurosurgery, Red Cross Hospital, 11526 Athens, Greece

## Abstract

*Objective*. Only limited data exists in terms of the incidence of intracranial bleeding (ICB) in patients with mild traumatic brain injury (MTBI). *Methods*. We retrospectively identified 3088 patients (mean age 41 range (7–99) years) presenting with isolated MTBI and GCS 14-15 at our Emergency Department who had undergone cranial CT (CCT) between 2002 and 2011. Indication for CCT was according to the “Canadian CT head rules.” Patients with ICB were either submitted for neurosurgical treatment or kept under surveillance for at least 24 hours. Pearson's correlation coefficient was used to correlate the incidence of ICB with age, gender, or intake of coumarins, platelet aggregation inhibitors, or heparins. *Results*. 149 patients (4.8%) had ICB on CCT. No patient with ICB died or deteriorated neurologically. The incidence of ICB increased with age and intake of anticoagulants without clinically relevant correlation (*R* = 0.11; *P* < 0.001; *R* = −0.06; *P* < 0.001). *Conclusion*. Our data show an incidence of 4.8% for ICB after MTBI. However, neurological deterioration after MTBI seems to be rare, and the need for neurosurgical intervention is only required in selected cases. The general need for CCT in patients after MTBI is therefore questionable, and clinical surveillance may be sufficient when CCT is not available.

## 1. Introduction

Mild traumatic brain injury (MTBI) is a major public health concern for both children and adults and represents 70–90% of all treated traumatic brain injuries [1, 2]. Prognosis after MTBI is a central issue in health care and is related to both the identification of individuals at risk of poor recovery and identification of modifiable risk factors and feasible treatment strategies [3]. Cranial CT without contrast (CCT) has become the gold standard to detect structural brain injury after traumatic brain injury. Several studies have been carried out to investigate both intracranial structural abnormalities and clinical symptoms and neurological deterioration after MTBI [4–8]. Stiell et al. reviewed more than 3000 patients undergoing CCT after MTBI in a prospective multicenter study and found clinically important brain injuries in 8% of all cases [8]. The authors proposed a score for the performance of CCT after MTBI, and this has become known as the Canadian CT head rules [8]. The indication for CCT in our department is in accordance with the Canadian CT head rules [8]. In case of an intracerebral bleed, a neurosurgeon has to be formally consulted. Patients with clinically unimportant brain injury undergo admission for 24 hours surveillance in the Emergency Department. Clinical surveillance comprises the assessment of GCS every 15 minutes for the first hour, every 30 minutes for the next 2 hours, and every hour for the following 21 hours [9]. However, it is not yet clear whether CCT is useful for all patients with MTBI and a Glasgow Coma Scale score (GCS) of 14-15 points, or whether clinical surveillance may be superior [2, 8, 10–12]. This study aimed at investigating the incidence of ICB, neurological deterioration, and possible risk factors, in a large consecutive group of European patients with isolated MTBI on admission.

## 2. Methods 

A retrospective study was conducted. Using our electronic patient records; we identified all patients presenting with isolated MTBI and GCS 14-15 at our Emergency Department (Level I trauma centre) between January 1, 2002 and December 31, 2011. All patients underwent CCT within less than 6 hours of their accident. Patients with GCS <14, missing or incomplete CCT data, or no clear history of trauma were excluded from the study. The indication for CCT was set according to the guidelines of the Canadian CT head rules [8]. Diagnostic findings from CCT were evaluated for intracranial bleeding (ICB). If structural brain injuries were found on CCT, patients were either submitted for further neurosurgical treatment or kept under surveillance for at least 24 hours, in order to assure early recognition of neurological deterioration related to MTBI. The type of ICB (epidural haematoma, subdural haematoma, subarachnoid haemorrhage, or intracerebral haematoma) was noted. Patients with anticoagulant medication (coumarins, platelet aggregation inhibitors, or heparins) were identified. The mechanisms of injury were noted. Statistical analyses included the determination of Pearson's correlation coefficient for age, gender, and the intake of coumarins, platelet aggregation inhibitors, or heparins with ICB [10]. A *P* value <0.05 was accepted as statistically significant. This study was approved by the institutional review board.

## 3. Results

**Figure 1:**
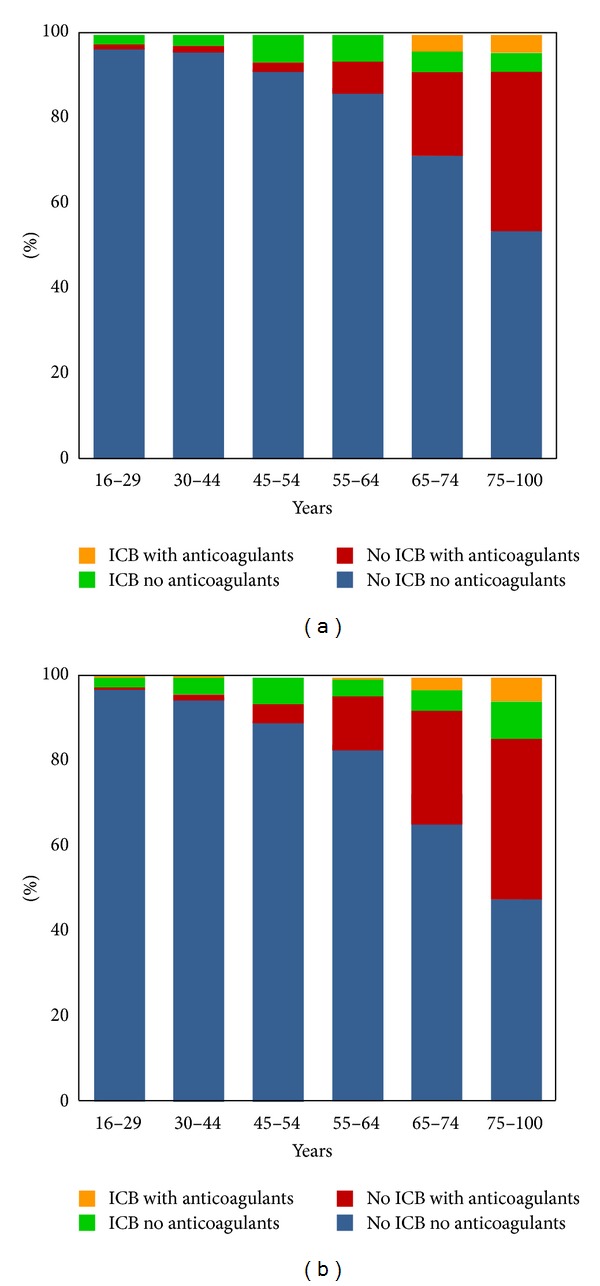
The distribution of intracranial bleeding (ICB) with and without anticoagulant drugs was shown for different age groups for male (a) and female (b) patients. The intake of anticoagulant drugs increased with increasing age. Anticoagulant drugs did not increase the risk for ICB.

A total of 3088 patients were identified. The mean age at the time of trauma was 41 ± 20.5 (range: 16–99) years. Sixty-one percent of all patients were male. 149 patients (4.8%) were found to have ICB on CCT (62% male). The different types of ICB (epidural, subdural haematoma, subarachnoid, and intracerebral haemorrhage) as well as gender distribution are shown in Table 1. No patient with ICB died or deteriorated neurologically after a minimum follow up 24 hours of clinical surveillance. The incidence of ICB after MTBI increased with age and the intake of anticoagulants. However, there was no clinically relevant correlation of ICB with age (*R* = 0.11; *P* < 0.001), gender (*R* = 0.002; *P* = 0.455), or the intake of anticoagulant drugs (*R* = −0.06; *P* < 0.001) (Figure 1). The main mechanisms of accident were falls, road accidents, violence, and sports.

## 4. Discussion

The present study aimed at investigating the incidence of ICB after MTBI. Of all 3088 patients, 149 (4.8%) presented with an ICB on CCT. There were no patients with neurological deterioration after 24 hours surveillance. None of the investigated risk factors showed a clinically relevant correlation with ICB.

There is still no broad agreement in terms of the need for CCT in patients with GCS 14-15 after MTBI [2, 8, 11–13]. Several studies have found similar results with regard to the incidence of ICB after MTBI [8, 14, 15]. Our data suggest that clinical surveillance seems to be sufficient and CCT may not be necessary in the acute phase, as none of the patients in this study presented with neurological deterioration. This would reduce unnecessary exposure to radiation and save radiology resources. A recent study showed that even admission and observation are not required in a subset of adult patients without risk factors for bleeding (e.g., anticoagulant therapy, intoxication, and concomitant injuries), suggesting a more liberal policy of early home monitoring [9]. On the other hand, minor bleeding found on CCT may guide the clinician with regard to mid- and long-term followup, since some patients may require elective neurological treatment due to secondary neurocognitive disorders related to MTBI. This may include postconcussion syndrome (PCS) [5, 7] or posttraumatic secondary epilepsy. Several studies showed considerable negative effects on the patient's ability to return to preinjury function, or to work or attend school after MTBI [16–21].

## 5. Limitations

This study has limitations. Due to the retrospective study design; this study includes only patients who had undergone CCT, thus neglecting those without CCT and GCS 14-15. For the latter, no followup was obtained and no outcome measure can be reported. However, in accordance with our in house policy, all patients with a history of trauma and fulfilling the Canadian CT hear rules were included. As this is a large patient series, we believe that this selection bias can be neglected. Furthermore, the intake of coumarins, platelet aggregation inhibitors, or heparins was not substantiated by specific laboratory measures such as INR or thrombin time. Therefore, no conclusions can be drawn with regard to the degree of anticoagulation. 

## 6. Conclusion

Our data show an incidence of 4.8% for ICB after MTBI. However, neurological deterioration after MTBI seems to be rare, and the need for neurosurgical intervention is only required in the selected cases. Anticoagulants, age, or gender do not seem to relevantly increase the risk of ICB. The general need for cranial CT in patients after MTBI is therefore questionable, and clinical surveillance may be sufficient when cranial CT is not available. Nevertheless, a CCT may be performed in patients whose comorbidities or concomitant injuries prohibit proper clinical/neurological assessment or surveillance.

## Figures and Tables

**Table 1:** Gender distribution and type of intracranial bleeding.

Characteristics	All patients with ICB	Female patients (%)	Male patients (%)
All ICB	149 (100%)	57 (38%)	92 (62%)
Epidural haematoma	9 (6%)	2 (1%)	7 (5%)
Subdural haematoma	25 (17%)	9 (6%)	16 (11%)
Subarachnoid haemorrhage	39 (26%)	21 (14%)	18 (12%)
Intracerebral haemorrhage	76 (51%)	25 (17%)	51 (34%)

ICB: intracranial bleeding. Percentages are shown for patients with ICB.
